# Regulation of Cholesterol Homeostasis by a Novel Long Non-coding RNA LASER

**DOI:** 10.1038/s41598-019-44195-2

**Published:** 2019-05-22

**Authors:** Chuanwei Li, Zhangxue Hu, Wen Zhang, Junyi Yu, Yang Yang, Zaicheng Xu, Hao Luo, Xiaoli Liu, Yukai Liu, Caiyu Chen, Yue Cai, Xuewei Xia, Xiaoqun Zhang, Da-zhi Wang, Gengze Wu, Chunyu Zeng

**Affiliations:** 1 Department of Cardiology, Daping Hospital, The Third Military Medical University, Chongqing, P.R. China; 2 Chongqing Institute of Cardiology, Chongqing, P.R. China; 3 Department of Pediatrics, Daping Hospital, The Third Military Medical University, Chongqing, P.R. China; 4 Department of Respiration, Xinqiao Hospital, The Third Military Medical University, Chongqing, P.R. China; 5 Department of Cardiology, Boston Children’s Hospital, Harvard Medical School, Boston, Massachusetts USA

**Keywords:** Dyslipidaemias, Dyslipidaemias, Genetics research

## Abstract

Genome-wide association studies (GWAS) have identified many genetic variants in genes related to lipid metabolism. However, how these variations affect lipid levels remains elusive. Long non-coding RNAs (lncRNAs) have been implicated in a variety of biological processes. We hypothesize lncRNAs are likely to be located within disease or trait-associated DNA regions to regulate lipid metabolism. The aim of this study was to investigate whether and how lncRNAs in lipid- associated DNA regions regulate cholesterol homeostasis in hepatocytes. In this study, we identified a novel long non-coding RNA in Lipid Associated Single nucleotide polymorphism gEne Region (LASER) by bioinformatic analysis. We report that LASER is highly expressed in both hepatocytes and peripheral mononuclear cells (PBMCs). Clinical studies showed that LASER expression is positively related with that of cholesterol containing apolipoprotein levels. In particular, we found that LASER is positively correlated with plasma PCSK9 levels in statin free patients. siRNAs mediated knock down of LASER dramatically reduces intracellular cholesterol levels and affects the expression of genes involved in cholesterol metabolism. Transcriptome analyses show that knockdown of LASER affects the expression of genes involved in metabolism pathways. We found that HNF-1α and PCSK9 were reduced after LASER knock-down. Interestingly, the reduction of PCSK9 can be blocked by the treatment of berberine, a natural cholesterol-lowering compound which functions as a HNF-1α antagonist. Mechanistically, we found that LASER binds to LSD1 (lysine-specific demethylase 1), a member of CoREST/REST complex, in nucleus. LASER knock-down enhance LSD1 targeting to genomic loci, resulting in decreased histone H3 lysine 4 mono-methylation at the promoter regions of HNF-1α gene. Conversely, LSD1 knock-down abolished the effect of LASER on HNF-1α and PCSK9 expressions. Finally, we found that statin treatment increased LASER expression, accompanied with increased PCSK9 expression, suggesting a feedback regulation of cholesterol on LASER expression. This observation may partly explain the statin escape during anti-cholesterol treatment. These findings identified a novel lncRNA in cholesterol homeostasis. Therapeutic targeting LASER might be an effective approach to augment the effect of statins on cholesterol levels in clinics.

## Introduction

Cardiovascular disease and stroke cause immense health and economic burdens worldwide^1^. Elevated low-density lipoprotein (LDL) cholesterol level is a significant risk factor for cardiovascular diseases. Many factors, including physical activity, diet, disease, and genetics, contribute to the variability of blood lipid levels. Genetic variations are heritable, non-modifiable risk factors and studies of genetic polymorphisms help to identify new targets for cholesterol therapy. Large-scale genome-wide association studies (GWAS) of genetic variations from unrelated individuals have shown that many single nucleotide polymorphisms (SNPs) loci are associated with blood lipids, accounting for ~10–12% of total trait variance^2^, especially the loci in 11q12 region were tightly associated with triglyceride levels^3,4^. However, the dilemma, when trying to explain the GWAS results, is that many of these variants (43%) lie in intergenic regions and hence their roles in regulating blood lipid levels remain largely unexplained. Moreover, some loci do not have genes involved in classical lipid metabolism pathways.

Recent studies revealed that gene regions, previously thought to be silent, can transcribe into a wide-range of functional transcripts designated as non-coding RNAs^5^. Long (>200 nt) non-coding RNAs (lncRNAs) are more likely to locate within disease or trait-associated regions from published GWAS catalog^6^. For example, the chromosome 9p21 locus was highlighted as the strongest genetic susceptibility locus for atherosclerosis, type 2 diabetes, cancer and glaucoma. This region encodes a novel long non-coding RNA, ANRIL (antisense non coding RNA in the INK4 locus). ANRIL specifically binds to two polycomb proteins (CBX7 in PRC1 and SUZ12 in PRC2) and has diverse roles in gene regulation^7^. Whether there are more functional lncRNAs in GWAS region that affect cholesterol metabolism are not known. We hypothesize that genes in lipid- associated GWAS regions could also encode a lncRNA that regulates lipid metabolism.

In the present study, we identified a novel lncRNA in lipid associated single nucleotide polymorphism locus (LASER) near SNP rs486394 in chromosome 11q12 region. Knock-down of LASER leads to reduction of cholesterol in HepG2 cells. LASER could bind to LSD1 directly; then regulate HNF-1a and proprotein convertase subtilisin/kexin 9 (PCSK9) expressions through REST/CoREST protein complex. LASER inhibition further reduced the intracellular cholesterol level caused by statin. We also found that the intracellular cholesterol regulated LASER expression. Taken together, the results from the current investigation revealed a mechanism in which LASER acts a feedback regulator of HNF-1α/PCSK9 and LXR dependent pathway to maintain cholesterol homeostasis in hepatocytes.

## Results

### Identification of LASER, a cholesterol associated lncRNA, in hepatocytes

**Figure 1:**
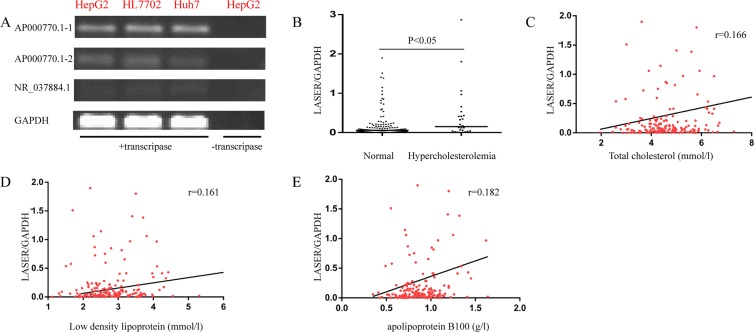
Identification of LASER in hepatocyte cell lines, and the association of LASER with cholesterol levels in PBMCs from patients. (**A**) The expressions of AP000770.1-1, AP000770.1-2 and NR_037884.1 in different cell lines were determined by semi-quantitative RT-PCR. The amplified products were visualized following 2% agarose gel electrophoresis. To verify the specificity of RT-PCR, one negative control is shown without reverse transcriptase. (**B**) LASER expression in PBMCs of the hypercholesterolemia (n = 26) and normal cholesterol subgroups (n = 149). Data are compared with the Mann–Whitney U test. (**C**–**E**) The Spearman correlation of LASER expression in PBMCs with the levels of total cholesterol (**C**), LDL-c (**D**) and apoB100 (**E**) in statin free individuals respectively (n = 175).

In order to find lncRNAs in genes related to lipid metabolism in the GWAS, we searched the lncSNP database and identified three lncRNAs near four SNP regions (Table 1). Among the three lncRNAs, ENSG00000253111 (TRIBAL) have been proven to have limited impact on either TRIB1 or lipid regulatory genes in human hepatocyte models^8^. We also found two putative transcripts (AP000770.1-1, AP000770.1-2) in ENSG00000237937 (AP000770.1) via the UCSC Genome Browser (http://genome.ucsc.edu/). Next, we detected the expression of lncRNAs in three well known cell models of hepatocytes (HepG2, HL7702 and Huh7) by Reverse Transcription-PCR (RT-PCR) analysis (Fig. 1A and Supplementary Fig. 1). We found that the expression of AP000770.1-1 was relatively higher than AP000770.1-2 or NR_037884.1 in hepatocytes. In reference to its gene region, we renamed it (AP000770.1-1) as LASER (long non coding RNA in Lipid Associated Single nucleotide polymorphism gEne Region). We assessed the coding potential of LASER by two prevailing tools, CPAT (Coding Potential Assessment Tool) and CPC (Coding Potential Calculator). For CPAT, the coding probability was 0.0126834, indicating that LASER was a non-coding transcript. For CPC, transcripts with scores of more than 1 were classified as “coding”, less than −1 as “non-coding”, and between −1 and 1 were classified as “weak non-coding”([−1, 0]) or “weak coding”([0, 1]), respectively. The CPC score of LASER was 0.0318, also indicating that LASER has very weak coding potential.

**Table 1:** LncRNAs in lipid associated GWAS regions.

SNP ID	Neighbor gene	LncRNA
rs6987702	TRIB1	ENSG00000253111
rs13721515	TRIB1	ENSG00000253111
rs486394	APO(A1/A4/A5/C3)	ENSG00000237937
rs2602836	ADH5	NR_037884.1

PBMCs from 175 individuals not receiving statin treatment were collected and the expression of LASER was quantified by qRT-PCR. The demographic characteristics of the enrolled individuals are listed in Supplementary Table 1. Overall, the mean total cholesterol (TC) and low density lipoprotein cholesterol (LDL-c) levels on admission were 4.6 mmol/l [SD, 1 mmol/l] and 2.8 mmol/l [SD, 0.7 mmol/l] respectively. Of the enrolled subjects, TC levels of 26 individuals met the criteria of hypercholesterolemia (TC ≥ 6.22 mmol/l). As shown in Fig. 1B, the LASER expression was significantly higher in the hypercholesterolemia subgroup (Fig. 1B). Next, we analyze the correlation of LASER expression and levels of TC, LDL-c and apoB100, a cholesterol trafficking apolipoprotein. We found that indeed the expression of LASER was positively associated with TC, LDL-c and B100 levels although such associations were weak (Fig. 1C–E). After adjusting the potential confounding variables (age, sex, TC, TG, LDL-c, HDL-c, apoA, apoB, uric acid, blood urea nitrogen, creatinine) by logistic regression analysis, we found that TC, LDL-c, and apoB100 levels remained independently and significantly associated with LASER expression (Table 2).

**Table 2:** Mutivariable logistic regression on LASER expression.

Variable	Standardized coefficiency	P value
TC	0.178	0.001*
LDL	0.366	0.000*
apoB	0.749	0.000*

n = 175, *P < 0.05.

### LASER regulates cholesterol metabolism via HNF-1a and PCSK9

**Figure 2:**
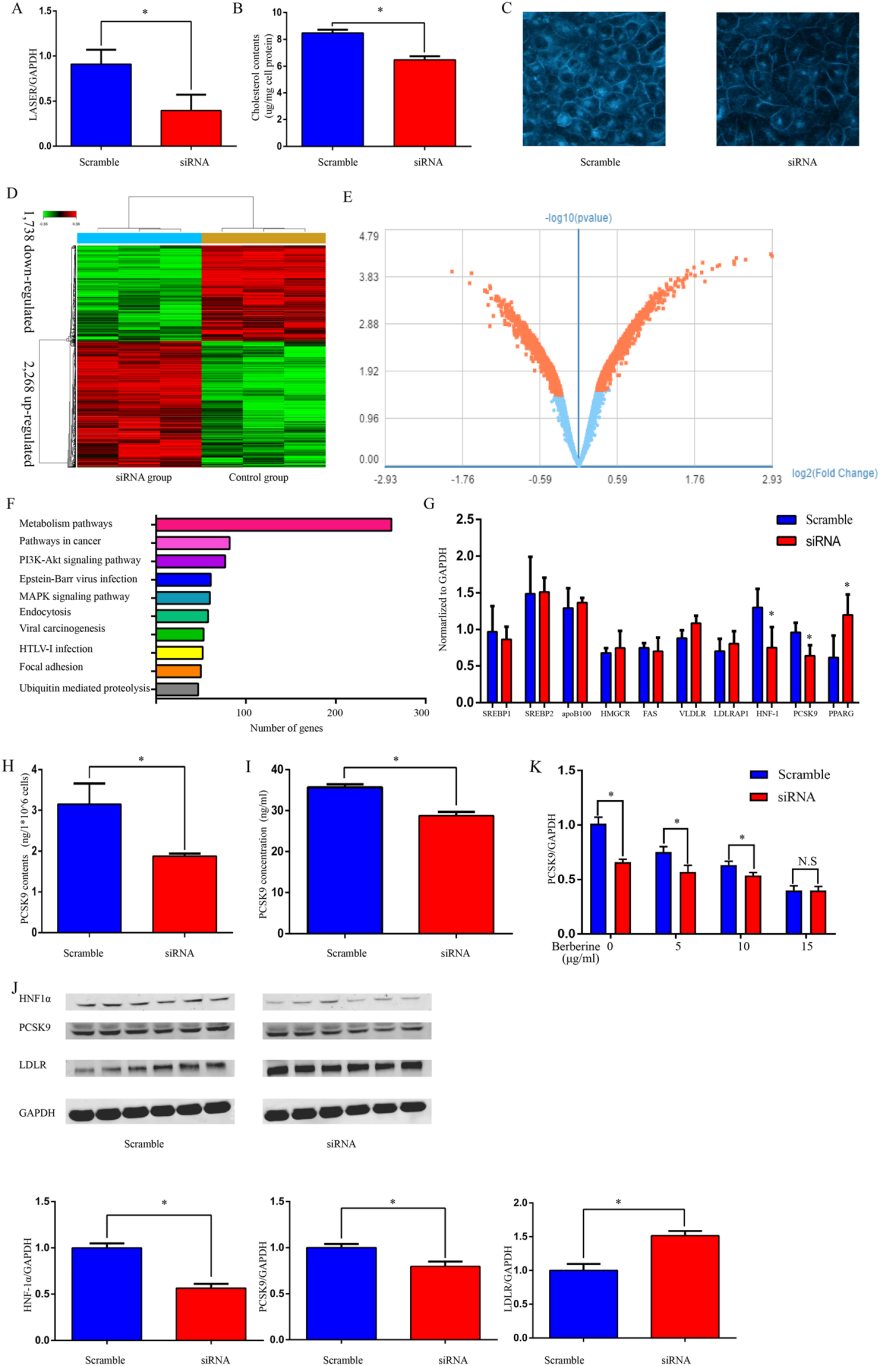
LASER regulate cholesterol metabolism through HNF-1α and PCSK9. (**A**) The expression of LASER was quantified by qRT-PCR in HepG2 cells incubated for 48 hrs with siRNA against LASER or scramble control (n = 6, *P < 0.05 versus control). (**B**,**C**) Effect of LASER siRNA on intracellular cholesterol level in HepG2 cells. After LASER knocked-down by siRNA, the intracellular cholesterol level was measured by Amplex red assay (**B**) (n = 6, *P < 0.05 versus control), or stained with filipin and observed by confocal laser-scanning microscopy (**C**). (**D**) Hierarchical clustering analyses of deregulated protein-coding genes (>1.2 folds change) after LASER knockdown in HepG2 cells. Up- and down-regulated genes are represented by red and green respectively (n = 3). (**E**) Volcano plot illustrating significant differential gene expression (mRNAs) changes (red marks) attributed to LASER knock-down. −Log10 (Benjamini–Hochberg-corrected p value) p < 0.05; Log2 (fold change) >1.2-fold (n = 3). (**F**) Pathway analysis indicating the number of deregulated genes (>1.2 folds change) after LASER knock-down. (**G**) Some of the key genes implicated in cell cholesterol metabolism were verified by qRT-PCR after LASER knocked-down. Gene expression were normalized to GAPDH (n = 6, *P < 0.05 versus control). (**H**,**I**) The concentration of PCSK9 was detected using ELISA methods in both cell lysate (**H**) and cell culture supernatants (**I**) of HepG2 cells treated with LASER siRNAs (50 nM) or scramble control (n = 6, *P < 0.05 versus control). (**J**) The protein levels of HNF-1α, PCSK9, LDLR and GAPDH were determined by western blotting after siRNA (50 nM) against LASER or scramble control (n = 6, *P < 0.05 versus control). (**K**) HepG2 cells were treated with different concentrations of berberine (0, 5, 10, 15 µg/ml) with or without LASER siRNA (50 nM) for 24 hrs. The expression levels of PCSK9 mRNAs were determined by qRT-PCR (n = 6, *P < 0.05 versus control, N.S: not significant).

To address the potential role of LASER in cholesterol metabolism, we decreased LASER expression in HepG2 cells using siRNA specifically directed against LASER. We achieved more than 50% knockdown of LASER expression (Fig. 2A). LASER knock-down significantly decreased the intracellular cholesterol levels (approximately 25%) as demonstrated by Amplex red assay (Fig. 2B). We performed Filipin staining and confirmed the reduction of free cholesterol in LASER siRNA treated group (Fig. 2C), further supporting the conclusion that LASER is involved in cholesterol metabolism in hepatocytes.

To define genome-wide global gene expression affected by the LASER regulatory pathway, we performed an unbiased microarray analysis using HepG2 cells treated with siRNA against LASER. A total of 4,024 differential expressed genes with more than 1.2 folds change were identified. Among them, 1,738 were down-regulated and 2,286 up-regulated when LASER was knocked down (Fig. 2D,E). Using pathway analysis, we found that the LASER is predominantly implicated in metabolism pathways (Fig. 2F). In addition, “pathways in cancer” and the “PI3K-Akt signaling pathway” were also dramatically altered when LASER was inhibited (Fig. 2F). Among the metabolism pathways, it was noticed that some crucial cholesterol metabolism associated genes, including PCSK9, were changed after the down-regulation of LASER. Among the metabolism pathways, we validated the microarray data by qRT-PCR in LASER siRNA group in HepG2 cells. The first seven genes which were very important candidate genes for cholesterol metabolism (SREBP1\SREBP2\apoB100\HMGCR\FAS\VLDLR\LDLRP) were not changed in the microarray data, and we confirmed it by qRT-PCR. It was noticed that some crucial cholesterol metabolism associated genes (HNF-1a\PCSK-9\PPARG), including PCSK9, were changed after the down-regulation of LASER (Fig. 2G). Besides the regulation by LASER of PCSK9 mRNA expression, we found that PCSK9 protein was reduced in HepG2 cell lysates and the cell culture supernatants of HepG2 cells (Fig. 2H,I) after knocked-down of LASER expression by siRNA. Consistent with the idea that LASER is required for the expression of PCSK9, finally, we found that LASER expression was positively correlated with plasma PCSK9 levels in statin free patients (Spearman correlation coefficient, 0.254, P < 0.05, n = 175).

PCSK9 has been reported to regulate LDLR expression by inducing intracellular degradation or post-translational regulation of the number of cell surface LDLR. PCSK9 inhibition by monoclonal antibodies exerts effective targets for reducing LDL-c levels^9,10^. The PCSK9 expression is regulated by a series of specialized transcription factors or cofactors in hepatocytes^11^. As shown in the qRT-PCR data (Fig. 2G), hepatocyte nuclear factor 1α (HNF-1α), a key transcriptional activator of PCSK9 gene expression, was also decreased after LASER knock-down. Similarly, the protein levels of both HNF-1α and PCSK9 were also decreased after LASER knock-down. By contrast, the expression of LDLR, which is the downstream target of HNF-1α and PCSK9, was up-regulated (Fig. 2J). These results suggest that LASER modulates the expression of lipid metabolism gene through the regulation of the HNF-1α/PCSK9 pathway.

Berberine is a natural cholesterol-lowering compound that can inhibit PCSK9 expression by inhibition of HNF-1α^12^. We tested whether berberine is involved in LASER-mediated regulation of lipid metabolism. We found that low dose berberine or LASER siRNA alone reduced PCSK9 expression, while administration of high dose berberine (15 µg/ml) to the LASER siRNA treated HepG2 cells abolished the inhibitory effects of LASER on PCSK9 expression (Fig. 2K), further supporting the view that HNF-1α is a key factor in the regulation of LASER on PCSK9 expression in hepatocytes.

### LASER binds to LSD1 directly

**Figure 3:**
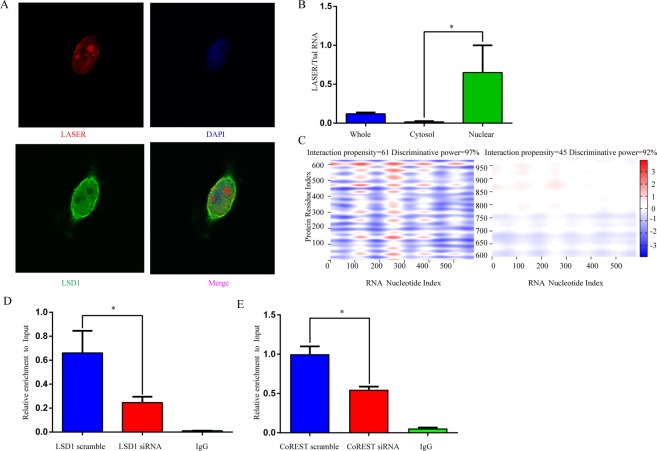
LASER binds to LSD1 directly. (**A**) LASER subcellular localization in HepG2 was detected by single molecule FISH using anti-sense probes against LASER (red) and LSD1 was determined by immunostaining with anti-LSD1 antibodies (green). Nuclei were counterstained with DAPI (blue) and detected by laser confocal microscopy. DAPI, 4,6-diamino-2-phenyl indole. (**B**) The expression of LASER was detected in different HepG2 cell fractions. Relative LASER abundance (defined as relative expression over total RNA) was measured by qRT-PCR (n = 3, *P < 0.05). (**C**) Predicted interaction of LASER and LSD1 protein by catRAPID analysis (http://service.tartaglialab.com/page/catrapid_group). (**D**) RNA immunoprecipitation experiments were performed using LSD1 antibody or IgG antibody as control in LSD1 siRNA (50 nM) or scramble control treated HepG2 cells, qRT-PCR was performed to detect pulled-down LASER (n = 3, *P < 0.05). (**E**) RNA immunoprecipitation experiments were performed using CoREST antibody or IgG antibody as control in CoREST siRNA (50 nM) or scramble control treated HepG2 cells, qRT-PCR was performed to detect pulled-down LASER (n = 3, *P < 0.05).

Next, we ask how LASER regulates the expression and function of HNF-1α and PCSK9. lncRNAs have been suggested to function either *in cis* or *in trans* [4–5, 9, 15]. We noticed that there was no change in the expression of LASER neighbor genes, the apoA1/C3/A4/A5 gene cluster at chromosome 11, after LASER knock-down, suggesting that LASER may act *in trans*. We found that LASER is more abundantly located in nucleus in HepG2 cells, using both Fluorescence *in situ* hybridization (FISH) and cellular fractionation studies (Fig. 3A,B). It has been suggested that lncRNAs in the nucleus mainly act as scaffold for recruiting the locus-specific chromatin-modifying enzymes to induce chromatin remodeling^13^. Using catRAPID (http://big.crg.cat/gene_function_and_evolution/services/catrapid), a widely-used prediction tool for protein-RNA interaction^14^, the nucleotides 100 to 400 of LASER was predicted to bind to the lysine (K)- specific demethylase 1A (LSD1) at amino acid residues 500–600. This prediction yielded the discriminative power of 97% (Fig. 3C). LSD1 is a histone demethylase encoded by the KDM1A gene which belongs to a member of the CoREST/REST protein complex. Using RNA Immunoprecipitation (RIP) assays, we confirmed the direct interaction between LASER and LSD1. LSD1 or CoREST knock-down by siRNA dramatically reduced protein available for binding, while the IgG negative control failed to retrieve LASER in HepG2 lysates (Fig. 3D,E**)**.

### LASER modulates the activity of LSD1 in regulating target gene expression

**Figure 4:**
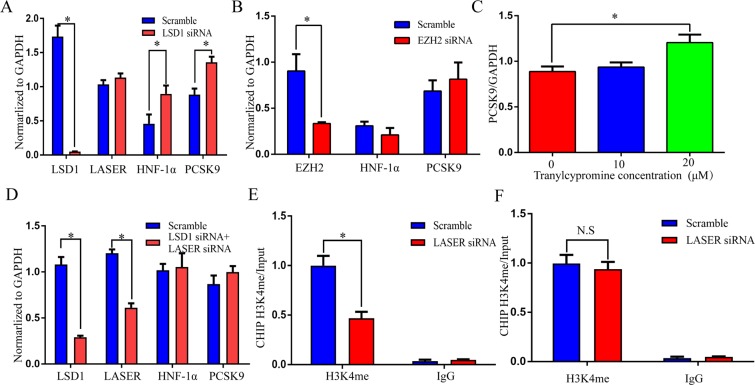
LASER modulates the activity of LSD1 in regulating target gene expression. (**A**) HepG2 cells were treated with LSD1 siRNA (50 nM). The expressions of LSD1, HNF-1α and PCSK9 were quantified in HepG2 cells using qRT-PCR after LSD1 knocked-down (n = 6, *P < 0.05). (**B**) HepG2 cells were treated with EZH2 siRNA (50 nM). The expressions of EZH2, HNF-1α and PCSK9 were tested in HepG2 cells using qRT-PCR after EZH2 knocked-down (n = 6, *P < 0.05). (**C**) The HepG2 cells were treated with LSD1’s inhibitor tranylcypromine (20 μM). The PCSK9 expression were quantified using qRT-PCR (n = 6, *P < 0.05 versus the control). (**D**) LSD1 and LASER were both knocked-down by siRNA (50 nM respectively) methods. The expressions of LSD1, LASER, HNF-1α, and PCSK9 were quantified by qRT-PCR (n = 6, *P < 0.05). (**E**) HepG2 cells were transfected with LASER siRNAs and scramble controls for 48 hrs. Then cells were lysed, and chromatin was collected for chromatin immunoprecipitation analysis. The H3K4 monomethylation status of HNF-1α gene promotor was analyzed by chromatin immunoprecipitation using an anti-K4H3me antibody or IgG controls, y-axis represent fold changes relative to input. (n = 6, *P < 0.05). (**F**) HepG2 cells were transfected with LASER siRNAs and scramble controls for 48 hrs. Then cells were lysed, and chromatin was collected for chromatin immunoprecipitation analysis. The H3K4 monomethylation status of PCSK9 gene promotor was analyzed by chromatin immunoprecipitation using an anti-K4H3me antibody or IgG controls, y-axis represent fold changes relative to input. (n = 6, *P < 0.05).

In order to examine whether LSD1 is involved in the regulation of lipid metabolism, we knocked down LSD1 by siRNA and found that the expression levels of HNF-1α and PCSK9 increased (Fig. 4A). In contrast, knocking down of EZH2, a member of the Polycomb Repressive Complex 2 (PRC2) that has been known to interact with lncRNAs, had no impact on HNF-1α or PCSK9 expression (Fig. 4B), demonstrating the specificity of LSD1 action. Moreover, administration of tranylcypromine, a LSD1’s demethylase activity inhibitor, raised PCSK9 expression in HepG2 cells (Fig. 4C). Intriguingly, the inhibitory effects of LASER siRNA on HNF-1α and PCSK9 expression were abolished when LSD1 was knocked-down. This observation suggests a role of LSD1 in the regulation of LASER on HNF-1α and PCSK9 expressions (Fig. 4D).

LSD1 (aka, KDM1 and AOF2) induces histone modifications primarily from H3K4 by removing the activating methylation marks from histones within their proximity. To test the hypothesis that LASER binds to LSD1 to modulate chromatin modifications at the promoter of HNF-1α gene, we performed ChIP-PCR to measure the levels of monomethylated lysines 4 on histone H3 (H3K4me) after LASER is knocked-down in hepatocytes. Indeed, LASER knock down specifically enhanced LSD1 mediated epigenetic modification, therefore resulting in decreased levels of active chromatin marks H3K4me (Fig. 4E). Meanwhile, LASER knocked-down didn’t affect H3K4me marks of PCSK9 promoter (Fig. 4F). Together, these studies demonstrate that lncRNA LASER functions in regulating gene expression by modulating the activity of epigenetic factor LSD1.

### Cholesterol feedback regulates LASER expression

**Figure 5:**
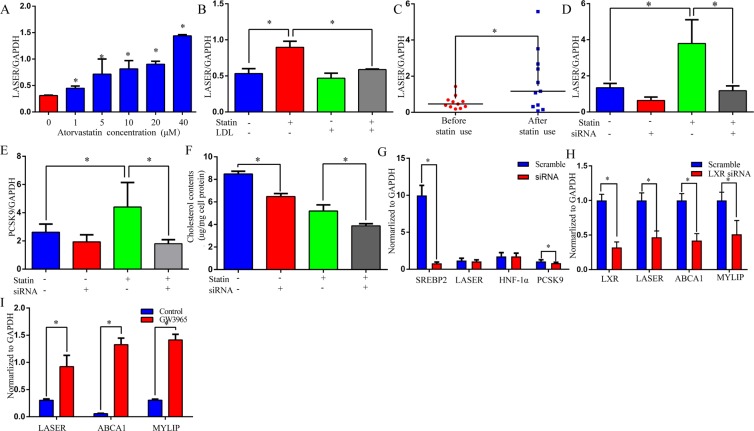
Cholesterol depletion feedback increase LASER expression. (**A**) HepG2 cells were treated with increasing concentrations of atorvastatin for 24 hrs and LASER expression was quantified by qRT-PCR (n = 6, *P < 0.05 versus the control). (**B**) HepG2 cells were incubated with 20 μM atorvastatin and 10 μM mevalonate to reduce cellular cholesterol content. The cells were then refed with or without LDL added to the medium to deliver cholesterol. The LASER expression was measured by qRT-PCR (n = 6, *P < 0.05). (**C**) Patients, who were treated with atorvastatin (20 mg/day) for 5 days had their blood collected before and after atorvastatin treatment. The LASER expression in PBMCs was quantified by qRT-PCR (n = 11, Data are compared with Mann–Whitney U test, *P < 0.05). (**D**,**E**) HepG2 cells were treated with 20 μM atorvastatin and/or siNRA (50 nM) against LASER for 24 hrs. The LASER (D) and PCSK9 (**E**) expressions were quantified by qRT-PCR methods (n = 6, *P < 0.05). (**F**) HepG2 cells were treated with LASER siRNA (50 nM) and/or atorvastatin (10 μM) for 24 hrs. The intracellular cholesterol level was measured by Amplex red assay. (**G**) The SREBP2 expression in HepG2 was knocked-down by siRNA (50 nM) for 48 hrs. The expressions of LASER, HNF-1a and PCSK9 were quantified by qRT-PCR methods (n = 6, *P < 0.05). (**H**) The LXR expression in HepG2 was knocked-down by siRNA (50 nM) for 48 hrs. The expressions of LASER and other two known LXR target genes (ABCA1 and MYLIP) were quantified by qRT-PCR methods (n = 6, *P < 0.05). (**I**) The expressions of LASER,ABCA1 and MYLIP were detected in HepG2 cells after incubated with liver X receptor agonist GW3965 (1 μM) for 24 hrs (n = 6, *P < 0.05).

Previous studies have found that cholesterol feedback regulates many genes involved in cholesterol homeostasis, the self-regulatory mechanism contributes to the maintenance of cholesterol levels in a normal range in most cases^15^. To investigate whether cholesterol regulates LASER expression in a feedback manner, we used atorvastatin, a member of the hydroxymethylglutaryl-coenzyme A reductase (HMG-CoA reductase) inhibitor statin family, to deplete cholesterol contents in HepG2 cells and examined the expression of LASER 24 hrs later. Indeed, LASER expression was strongly induced by atorvastatin incubation in a dose-dependent manner (Fig. 5A). Most importantly, this induction was efficiently reversed by the add-back of cholesterol through LDL administration, demonstrating the specificity of such induction (Fig. 5B).

We next asked whether cholesterol could control LASER expression level in patients in a similar feedback pattern. We detected LASER expression in PBMCs of 11 CAD patients who had been prescribed with statin treatment. We found that while the administration of statin reduced cholesterol level, it increased LASER expression level simultaneously (Fig. 5C). The statin induced LASER up-regulation can subsequently increase PCSK9 level and decrease the hepatic clearance of LDL from the circulation, which may limit the therapeutic effect of statins. We therefore asked whether a combination of LASER knock-down and statin treatment could achieve better reduction of PCSK9 and cholesterol levels. As expected, statin treatment alone substantially reduced cholesterol levels and slightly increased PCSK9 levels, however, LASER knockdown combined with statin treatment can further reduced the PCSK9 and cholesterol levels (Fig. 5D–F). These results suggest that combination of statin treatment with LASER inhibition could become a more effective therapy for lowering cholesterol.

It is well recognized that both sterol response element binding proteins (SREBP2) and liver X receptor (LXR) play a key role in cellular response to sterols. The knock-down of SREBP2 expression by siRNAs did not affect LASER or HNF-1α expression and slightly decrease PCSK9 expression (Fig. 5G). However, LXR knock-down significantly reduced expressions of LASER and another two known LXR target genes (ABCA1 and MYLIP) (Fig. 5H). Moreover, when HepG2 cells were incubated with 1 μM GW3965, a selective synthetic LXR agonist, the expression of LASER, ABCA1 and MYLIP increased significantly, despite the extent of ABCA1 and MYLIP up-regulation being more pronounced than LASER (Fig. 5I).

## Discussion

**Figure 6:**
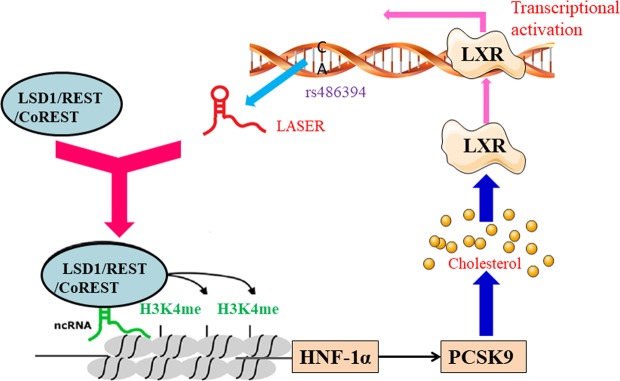
Schematic representation of a working model by which LASER regulates cholesterol metabolism. Chromosome 11q12 encodes a novel non-coding RNA-LASER that is involved in lipid metabolism. LASER binds to LSD1 protein in REST/coREST complex and regulate LSD1-mediated H3K4me demethylation in HNF-1α gene promoter and subsequently regulate PCSK9 expression and cholesterol metabolism in hepatocytes. Statin inhibits cholesterol synthesis but increases LASER expression by LXR receptors. Our study revealed a cholesterol-mediated negative feedback of LASER expression in hepatocytes.

In the current study, we identified a novel lnc-RNA, LASER from GWAS- reported lipid associated SNP regions. The LASER expression is positively associated with cholesterol levels both *in vivo* and *in vitro*. LASER knock-down significantly reduced the intracellular cholesterol content by inhibition of the expression of PCSK9. LASER binds to LSD1 and represses LSD1-mediated histone demethylation at the HNF-1α gene promoter in the nucleus. The feedback regulation of LASER expression by cholesterol uncovered a network of cholesterol homeostasis maintenance involving non-coding RNA, transcription factors, and protein coding genes (Fig. 6).

In recent years, lncRNAs have been continuously discovered at an unprecedented pace to be actively involved in cholesterol homeostasis maintenance^16–18^. For example, lnc-HC negatively regulates cholesterol metabolism through physical interaction with hnRNPA2B1 and decreases two key genes (ABCA1 and Cyp7a1) in cholesterol metabolism^19^. LncRNA LeXis transcriptional regulates cholesterol biosynthesis genes in the mouse liver and alters the cholesterol content^20^. The expression of another lncRNA, RP1-13D10.2, reflects the response to statin therapy; the cholesterol feedback regulates RP1-13D10.2 expression through SREBP2 and LXR receptor-dependent pathways^21^. However, elucidation of the molecular basis of lncRNA function and verification of its effect on cholesterol in patients is a major challenge. Our present study found that LASER, another important cholesterol response lncRNA identified from published GWAS, regulates cholesterol homeostasis by affecting the epigenetic state of the HNF-1α chromatin and PCSK9 expression in hepatocytes. This finding, along with other aforementioned reports, identifies a complex network of cholesterol homeostasis-regulatory mechanisms, involving a class of epigenetic regulatory lncRNAs in hepatocytes.

As shown in the results, LASER is mainly localized in the nucleus. A number of reports support a prevailing mechanism for nuclear lncRNA to directly interact with and organize histone writers, readers, and modifiers to regulate gene transcription. For example, lncRNA Morrbid recruits and promotes accumulation of EZH2 protein in PRC2 complex to the Bcl2l11 promoter and in turn increase the repressive histone modifications (H3K27me3)^22^. We used bioinformatic methods to identify LSD1 as the mediator of LASER action on HNF-1α and verify the interaction by RIP and ChIP-qPCR methods. LSD1, a flavin-dependent lysine 4 of histone protein H3 (H3-Lys4) demethylase, forms a protein complex with CoREST to recognize the nucleosomal substrate and exert repressive activity through synergistic histone deacetylation and Lys4 demethylation^23^. Given the fact that cholesterol metabolism modulation forms a complex and precise network involving many genes and transcriptional factors, our hypothesis of LSD1-mediated LASER regulation on PCSK9 may be one of many potential pathways, the comprehensive molecular mechanisms of which are not yet elucidated. Moreover, the molecular structural basis of LASER interacting with LSD1 requires further investigation.

The cholesterol-mediated negative feedback on LASER expression is not unexpected. The wide existence of positive and negative feedback regulatory networks ensure that metabolites (e.g., cholesterol, glucose and sodium) are maintained in normal ranges, which are required for the basic physiological demands and prevent excessive lipid accumulation in organs. However, this principle creates a new dilemma when high intensity statin therapy is required to further decrease the LDL-c levels to attain the increasingly strict goals recommended by current guidelines. One of the most important factors is the ability of statins to up-regulate the PCSK9 expression, which reduces the statin-mediated lowering of cholesterol levels, in a SREBP2-dependent pathway. PCSK9 inhibition by monoclonal antibodies, so far, is the most effective means to reduce further serum LDL-c levels. However, the degree of LDL-c reduction is dose-dependent with considerable inter-individual variation. High-risk patients for arteriosclerotic cardiovascular disease who have less-than-anticipated responses or are intolerant to guideline-recommended statin therapy may consider combination therapy. In this study we demonstrate that LASER inhibition during statin use in HepG2 cells can partially compensate for the secondary increase in PCSK9 expression, making LASER a potential target for translational research. The LASER expression after statin use could be a biomarker for statin response.

In summary, our work identifies a novel lncRNA, LASER, in hepatocytes from GWAS-reported catalog and uncovers its role in cholesterol homeostasis. LASER is not only involved in the complex regulatory network of cholesterol metabolism, but also provides potential new therapeutic targets to further reduce cholesterol levels in combination with statins.

## Methods

### Study subjects

The study subjects were recruited from Daping Hospital from February 2014 to November 2015. All subjects were unrelated individuals of Han Chinese from the southwest region of China. The subjects who were on lipid lowering drugs (statins) were excluded from the study. Patients underwent complete medical history and examination; basic clinical, biochemical, and demographic variables were collected under fasting state (The basic characteristics are shown in Supplementary Table 1). Another group, comprised of 11 patients, was enrolled in the statin response research, the blood samples were collected before and 5–7 days after statin therapy respectively (Supplementary Table 2). Blood samples and peripheral blood monocytes (PBMCs) were obtained and prepared according to previous reports^24,25^. A written informed consent for this study was obtained from all the participants. All study protocols were written in accordance with the Declaration of Helsinki and were approved by the Medical Ethics Committee of Daping Hospital.

### Bioinformatic analysis

The database of the lipid-associated SNPs with human long non-coding RNAs (http://bioinfo.hrbmu.edu.cn/LincSNP, version 1.0) was used in this study^26^. The protein-coding potential of LASER sequence was evaluated by Coding Potential Assessment Tool (CPAT, http://lilab.research.bcm.edu/cpat/index.php)^27^ and Coding Potential Calculator (CPC 2.0, http://cpc2.cbi.pku.edu.cn/)^28^.

### Cell culture

Human hepatocellular carcinoma cell lines (HepG2) were purchased from the Institute of Biochemistry and Cell Biology of the Chinese Academy of Sciences (Shanghai, China). The cells were cultured in Dulbecco’s Modified Eagle Medium (DMEM, Gibco, NY, USA), supplemented with 10% fetal bovine serum (FBS, Gibco) as well as 100 U/ml penicillin and 100 μg/ml streptomycin (Invitrogen, CA, USA) and maintained at 37 °C with 5% CO2.

Small double-strand interference RNAs (siRNAs) for LASER (CCUCUUCUAAGCUCUUUAUTT), LSD1 (GAAGCTACATCTTACCTTA), EZH2 (GCTGAAGCCTCAATGTTTA), SREBP2 (CAAGGAGAGTCTATACTGT), CoREST(AAGAUUGUCCCGUUCUUGACU), LXRα (CTGCCCAGCAACAGTGTAA) and control scramble siRNA (UUCUCCGAACGUGUCACGUTT) were synthesized by GenePharma (Shanghai, China).

For RNAi assay, HepG2 cells were seeded at a density of 3.0 × 10^5^ cells per well in a 6-well plate, when the density of cell reach to 70–90% the next day, transfection was performed. After the density of the cells reached to 70–90%, transfection was performed by incubating the siRNA with Lipofectamine RNAiMAX in Opti-MEM for 30 min at room temperature to allow siRNA-lipid complexes to form. Then, the mixed reagents were added to the target cells for a final concentration of 50 nM siRNA. After incubation with siRNA for 48 hrs, the cells were harvested for the experiments.

### RNA isolation and qRT-PCR analysis

Total RNAs were isolated using RNeasy kit (Qiagen, Hilden, Germany), following manufacturer’s instructions, and quantified by spectrophotometry (Nanodrop 2000). Reverse transcription (RT) was performed using M-MLV Reverse Transcriptase (Invitrogen). Both random hexamers (Applied Biosystems) and oligo-dT (Invitrogen) were used for reverse RT reactions. Real time quantitative PCR (qRCR) was performed using SYBR green master mix (Takara) following manufacturer’s instructions. GAPDH was used for normalization. The raw Ct values for GAPDH was about 15 and LASER was about 30. PCR primer sequences are listed in Supplementary Table 3.

### RNA library preparation and pathway analysis

HepG2 cells were incubated for 48 hrs with LASER siRNA or scramble controls (n = 3). Total RNA was isolated as previous stated. Unbiased genome-wide transcriptome profiling was performed using the HUGENE 1.0 ST array (Affymetrix), which interrogates a total of 17,000 Refseq transcripts. Arrays were probed with cDNAs derived from HepG2 cells treated with siRNA against LASER or controls. The microarray hybridization was performed based on the manufacturer’s standard protocols. The Affymetrix Expression Console (version 1.2.1) implementation of robust microarray analysis (RMA) was used for quantile normalization and background correction. All gene level files were imported into Affymetrix Expression Console (version 1.2.1) and normalized by the RMA algorithm. A random variance model (RVM) t-test was applied to discriminate differentially expressed mRNAs between siRNA group and controls. After false-discovery rate (FDR) analysis, differentially expressed genes were identified only if p value < 0.05 and over 1.2 folds or less than 0.8 fold dysregulated. Hierarchical clustering was performed using EPCLUST. Pathway analysis was used to find out the significant pathway of the differential genes according to KEGG, Biocarta and Reatome. Still, we turn to the Fisher’s exact test and *χ*^2^ test to select the significant pathway, and the threshold of significance was defined by P-value and FDR.

All primary microarray data were submitted to NCBI GEO database. GEO accession number is GSE86433 and all data can be downloaded from http://www.ncbi.nlm.nih.gov/geo/query/acc.cgi?acc=GSE86433.

### Filipin labeling of membrane cholesterol

Filipin binds to free cholesterol and is widely used to analyze the sequestration of unesterified cholesterol in cells^29^. HepG2 cells were fixed with 4% paraformaldehyde in phosphate buffered solution (PBS) for 15 min, washed with PBS and incubated at room temperature for 2 hrs in the dark with a stain solution containing 100 μg/ ml filipin (Sigma) in PBS. The cells were washed thrice with PBS for 5 min. Slides were sealed with cover slips. After 12 hrs of drying, fluorescence pictures were taken with an Olympus AX70 laser confocal microscopy (Olympus, Tokyo, Japan).

### Cholesterol assay

HepG2 cells treated with LASER siRNA were plated in 6-well cell culture plates and maintained in DMEM containing 5% lipoprotein-deficient serum (LPDS, Sigma) from calf and 10 μM mevalonate (Sigma) for 24 hrs. Then the cells were homogenized in ice-cold lysis buffer (PBS with 1% NP40, 0.5% sodium deoxycholate, 0.1% SDS, 1 mmol/L EDTA, 1 mM EGTA, 1 mM PMSF, 10 ng/ml aprotinin, and 10 ng/ml leupeptin). Cholesterol was detected using an Amplex red Cholesterol Assay kit (Molecular Probes) relative to a dilution series of cholesterol standards as specified. Briefly, the samples were diluted in reaction buffer, after which an equivalent volume of Amplex Red working solution (300 µM Amplex Red, 2 U/ml cholesterol oxidase, 2 U/ml cholesterol esterase, and 2 U/ml horseradish peroxidase) was added. The samples were incubated at 37 °C for 30 min and absorbance was measured at 590 nm using a microplate auto-reader (Varioskan Flash, Thermo Scientific). Cholesterol values were normalized to protein content as measured by the modified Lowry technique. In the dose response to statin experiments, atorvastatin (Sigma) was dissolved in DMSO and added to the HepG2 cell culture dishes at various concentrations (0, 1, 5, 10, 20 and 40 μM) for 24 hrs before cell harvest.

### ELISA detection of PCSK9

HepG2 cells (~1 × 10^6^ cells/well) were transfected with LASER siRNAs for 48 hrs and the cell culture supernatants were collected by centrifugation. The cells were then lysed in cell lysis buffer (R&D Systems) with periodic vortexing for 30 min. Fifty μl of sample or control conjugated with polyclonal antibody specific for human PCSK9 were added to the 96-well and incubated for 2 hrs at room temperature. PCSK9 levels were quantified by a high-sensitivity, quantitative sandwich enzyme immunoassay (Quantikine ELISA, R&D Systems). The absorbance at 450 nm was measured with a microplate auto-reader. The minimum detectable concentration was 0.096 ng/ml for this assay.

### Western blot

The proteins were prepared by homogenizing HepG2 cells in RIPA buffer ((1% v/v Triton X-100, 0.1% w/v sodium dodecyl sulfate, 0.5% w/v sodium deoxycholate, 10 mM sodium phosphate, pH 7.5, 5 mM EDTA, 5 mM EGTA, 100 mM sodium chloride, 1 mM PMSF, 20 M leupeptin, 20 mM methionine, and 1 mM cysteine)) containing a protease inhibitor cocktail on ice. The proteins were resolved on a 5% stacking, 8% running gel and transferred onto a nitrocellulose membrane. The membranes were blocked overnight with 5% nonfat dry milk in PBS with 0.1% Tween 20 and probed with primary antibodies overnight at 4 °C, followed by incubation with secondary antibodies for 90 min at room temperature. The membrane-bound antibodies were visualized using horseradish peroxidase-conjugated secondary antibodies (1:12,000) and the Odyssey Infrared Imaging System (Li-Cor Bioscience, Bad Homburg, Germany). Normalization was performed by blotting the same samples with an antibody against GAPDH. The primary antibodies were: PCSK9 (Abcam), LDLR (Abcam) and HNF1α (Abcam), each used at a dilution of 1:1000.

### Subcellular fractionation

The separation of nuclear and cytosolic fractions was performed using the PARIS Kit (Life) according to the manufacturer’s instructions.

### RNA fluorescence *in situ* hybridization (FISH) and immunofluorescence

To detect cellular distribution of LASER, HepG2 cells were rinsed briefly in PBS and then fixed in 4% formaldehyde in PBS for 10 min at room temperature. The cells were permeabilized in PBS containing 0.2–0.5% Triton X-100 and 5 mM vanadyl ribonucleoside complex (10 mM) on ice for 5 min, washed in PBS 3 × 10 min and rinsed once in 2 × saline-sodium citrate buffer. Rabbit monoclonal antibody against human LSD1 (Abcam) was added to the cells and incubated for more than 12 hrs at 4 °C. After incubation with rhodamine-conjugated goat anti-rabbit IgG (1:200 dilution) for 30 min, the cells were washed again with PBS. Hybridization was carried out using DNA probe sets (Ribo) in a moist chamber at 37 °C overnight according to the protocol. After RNA-FISH and immunol staining, cell nuclei were stained with 4′, 6-diamidino-2-phenylindole (DAPI) again. Cells were observed with a laser confocal microscopy (Olympus).

### RNA immunoprecipitation

RNA immunoprecipitation (RIP) experiments were performed with the Magna RIP RNA-Binding Protein Immunoprecipitation Kit (Millipore, Darmstadt, Germany) following the manufacturer’s instructions. Briefly, 2 × 10^7^ HepG2 cells treated with LSD1 siRNA or control were lysed in complete RIP lysis buffer and incubated on ice. Ten micrograms of rabbit anti-KDM1/LSD1 polyclonal antibody (Abcam, Cambridge, UK) or rabbit IgG antibody was used in each RIP reaction. The immunoprecipitate was further digested with proteinase K and RNA was isolated by phenol-chloroform-isoamyl alcohol (125:24:1, pH = 4.3) for qRT-PCR quantification.

### Chromatin immunoprecipitation analysis

Chromatin immunoprecipitation (ChIP) assays of HepG2 cells were performed using an EZ-Zyme Chromatin Prep kit and Chromatin Immunoprecipitation kit (Millipore) according to the manufacturer’s instruction. In brief, HepG2 cellls were cross-linked using a final fresh formaldehyde concentration of 1% at room temperature for 5 min. The reaction was quenched with the addition of glycine. The cell lysates were snap-frozen in a liquid N_2_ bath and then the pelleted nuclei were further cleaved in reaction buffer containing 1 U EZ-Zyme enzymatic cocktail and protease inhibitor cocktail. Cleaved chromatins were incubated overnight at 4 °C with control IgG, 5 μg of anti-Histone H3 (mono methyl K4) antibody (H3K4me, Abcam). Protein G agarose was added for 1 h with rotation. After incubation, the Protein G agarose-antibody/chromatin complex was serially washed with low salt wash buffer, high salt wash buffer, LiCL wash buffer, and finally TE buffer. Reverse cross-linking was performed at 65 °C overnight. After further purification with RNAse A and proteinase K, the DNA was extracted using a phenol-chloroform phase lock tube. qRT-PCR method was used to amplify the HNF-1α and PCSK9 gene promotor, primer sequences are listed in Supplementary Table 3.

### Statistical analysis

Data are expressed as mean ± standard error unless other stated. Two-tailed Student’s t test or ANOVA was used to assess differences between two groups or among more than two groups respectively using the Statistical Package for Social Sciences software package (SPSS Statistics version 18.0, USA). In a situation when the variable did not follow a normal distribution, we used the non-parametric Mann–Whitney U test or Kruskal-Wallis test. Univariate correlations between LASER expression and other continuous variables were assessed using Spearman’s correlation. Multiple logistic regression analysis was used to evaluate the potential bias factors which may contribute to LASER expression adjusting for potential clinical and biochemical factors. P < 0.05 was considered significant.

## Supplementary information

Dataset 1

## References

[1] Moran AE (2014). Temporal trends in ischemic heart disease mortality in 21 world regions, 1980 to 2010: the Global Burden of Disease 2010 study. Circulation..

[2] Willer CJ (2008). Newly identified loci that influence lipid concentrations and risk of coronary artery disease. Nat Genet..

[3] Teslovich TM (2010). Biological, clinical and population relevance of 95 loci for blood lipids. Nature..

[4] Aulchenko YS (2009). Loci influencing lipid levels and coronary heart disease risk in 16 European population cohorts. Nat Genet..

[5] Viereck J (2016). Long noncoding RNA Chast promotes cardiac remodeling. Sci Transl Med..

[6] Castellanos-Rubio A (2016). A long noncoding RNA associated with susceptibility to celiac disease. Scienc..

[7] Chen D (2014). ANRIL inhibits p15(INK4b) through the TGFβ1 signaling pathway in human esophageal squamous cell carcinoma. Cell Immunol..

[8] Douvris A (2014). Functional analysis of the TRIB1 associated locus linked to plasma triglycerides and coronary artery disease. J Am Heart Assoc..

[9] Ai D (2012). Regulation of hepatic LDL receptors by mTORC1 and PCSK9 in mice. J Clin Invest..

[10] Li C (2015). Efficiency and safety of Proprotein Convertase Subtilisin/Kexin 9 monoclonal antibody on hypercholesterolemia: A meta-analysis of 20 randomized controlled trials. J Am Heart Assoc..

[11] Schulz R, Schlüter KD, Laufsm U (2015). Molecular and cellular function of the proprotein convertase subtilisin/kexin type 9 (PCSK9). Basic Res Cardiol..

[12] Dong B, Li H, Singh AB, Cao A, Liu J (2015). Inhibition of PCSK9 transcription by berberine involves down-regulation of hepatic HNF1α protein expression through the ubiquitin-proteasome degradation pathway. J Biol Chem..

[13] Tsai MC (2010). Long noncoding RNA as modular scaffold of histone modification complexes. Science..

[14] Bellucci M, Agostini F, Masin M, Tartaglia GG (2011). Predicting protein associations with long noncoding RNAs. Nat Methods..

[15] Ota T (2015). Impact of the statin escape phenomenon on long-term clinical outcomes in patients with acute myocardial infarction: Subgroup analysis of the Nagoya Acute Myocardial Infarction Study (NAMIS). Atherosclerosis..

[16] Qin W (2016). A long non-coding RNA, APOA4-AS, regulates APOA4 expression depending on HuR in mice. Nucleic Acids Res..

[17] Halley P (2014). Regulation of the apolipoprotein gene cluster by a Long noncoding RNA. Cell Rep..

[18] Li P (2015). A liver-enriched long non-coding RNA, lncLSTR, regulates systemic lipid metabolism in mice. Cell Metab..

[19] Lan X (2016). A novel long noncoding RNA Lnc-HC binds hnRNPA2B1 to regulate expressions of Cyp7a1 and Abca1 in hepatocytic cholesterol metabolism. Hepatology..

[20] Sallam T (2016). Feedback modulation of cholesterol metabolism by the lipid-responsive non-coding RNA LeXis. Nature..

[21] Mitchel K (2016). RP1-13D10.2 is a novel modulator of statin-induced changes in cholesterol. Circ Cardiovasc Genet..

[22] Kotzin JJ (2016). The long non-coding RNA Morrbid regulates Bim and short-livedmyeloid cell lifespan. Nature..

[23] Pilotto S (2015). Interplay among nucleosomal DNA, histone tails, and corepressor CoREST underlies LSD1-mediated H3 demethylation. Proc Natl Acad Sci USA.

[24] Li C (2016). Zonulin regulates intestinal permeability and facilitates enteric bacteria permeation in coronary artery disease. Sci Rep..

[25] Wu G (2014). LincRNA-p21 regulates neointima formation, vascular smooth muscle cell proliferation, apoptosis, and atherosclerosis by enhancing p53 activity. Circulation..

[26] Ning Shangwei, Zhao Zuxianglan, Ye Jingrun, Wang Peng, Zhi Hui, Li Ronghong, Wang Tingting, Li Xia (2014). LincSNP: a database of linking disease-associated SNPs to human large intergenic non-coding RNAs. BMC Bioinformatics.

[27] Wang L (2013). CPAT: Coding-Potential assessment tool using an alignment-free logistic regression model. Nucleic Acids Res..

[28] Kang Y (2017). CPC2: a fast and accurate coding potential calculator based on sequence intrinsic features. Nucleic Acids Res..

[29] Chu BB (2015). Cholesterol transport through lysosome-peroxisome membrane contacts. Cell..

