# Supplementary material for: Regulation of Cholesterol Homeostasis by a Novel Long Non-coding RNA LASER
Source: Sci Rep. 2019 May 22;9:7693. doi: 10.1038/s41598-019-44195-2 (PMC6531449; doi:10.1038/s41598-019-44195-2)
Supplement: Supplementary file 1 — Dataset 1 [file 41598_2019_44195_MOESM1_ESM.docx]

**Regulation of Cholesterol Homeostasis by a Novel Long Non-coding RNA LASER**

Chuanwei Li^1,2^, Zhangxue Hu^3^, Wen Zhang^4^, Junyi Yu^1,2^, Yang Yang^1,2^, Zaicheng Xu^1,2^, Hao Luo^1,2^, Xiaoli Liu^1,2^, Yukai Liu^1,2^, Caiyu Chen^1,2^, Yue Cai^1,2^, Xuewei Xia^1,2^, Xiaoqun Zhang^1,2^, Da-zhi Wang^5^, Gengze Wu*^1,2^, Chunyu Zeng*^1,2^

^1^From the Department of Cardiology, Daping Hospital, The Third Military Medical University, Chongqing, P.R. China; ^2^Chongqing Institute of Cardiology, Chongqing, PR China. ^3^Department of Pediatrics, Daping Hospital, The Third Military Medical University, Chongqing, P.R. China;

^4^Department of Respiration, Xinqiao Hospital, The Third Military Medical University, Chongqing, P.R. China; ^5^Department of Cardiology, Boston Children's Hospital, Harvard Medical School, Boston, Massachusetts, USA.

#These authors contributed equally to this work

*Correspondence to:

Chunyu Zeng, MD, PhD

Department of Cardiology,

Daping Hospital,

The Third Military Medical University,

Chongqing City, P.R. China.

E-mail: [chunyuzeng01@163.com](mailto:chunyuzeng007@163.com)

Fax: 0086-02368757801

Tel: 0086-02368757801

Gengze Wu, MD, PhD

Department of Cardiology,

Daping Hospital,

The Third Military Medical University,

Chongqing City, P.R. China.

E-mail: wugengze@163.com

**Supplementary** **Table 1**. **Demographic characteristics of the study subjects**

| **Variable** | **Value(mean±standard deviation)** |
| --- | --- |
| N (M/F) | 88/87 |
| Age (year) | 61.1±12.1 |
| BMI (kg/m^2^) | 23.7±3.5 |
| SBP (mmHg) | 123.7±17.2 |
| DBP (mmHg) | 73.9±9.3 |
| Total cholesterol (mmol/l) | 4.6±1.0 |
| Triglyceride (mmol/l) | 1.3 (1.0 to 2.1) * |
| LDL-c (mmol/l) | 2.8±0.7 |
| HDL-c(mmol/l) | 1.2±0.3 |
| apoA (g/l) | 1.3±0.2 |
| apoB100 (g/l) | 0.9±0.2 |

* Data are expressed as median (interquartile range)

**Supplementary** **Table 2**. **Demographic characteristics of the statin treatment groups**

| **Variable** | **Value (mean±standard deviation)** |
| --- | --- |
| N (M/F) | 7/4 |
| Age (year) | 62.4±6.5 |
| BMI (kg/m^2^) | 24.8±3.6 |
| SBP (mmHg) | 124.4±13.1 |
| DBP (mmHg) | 72.5±8.6 |
| Total cholesterol (mmol/l) | 4.9±0.7 |
| Triglyceride (mmol/l) | 1.4 (0.9 to 2.3) * |
| LDL-C (mmol/l) | 2.9±0.5 |
| HDL-C (mmol/l) | 1.3±0.4 |
| apoA (g/l) | 1.4±0.4 |
| apoB100 (g/l) | 0.9±0.2 |

* Data are expressed as median (interquartile range)

**Supplementary Table 3 PCR primers used in this study**

| Gene | Forward primer（5'-3'） | Reverse primer（5'-3'） |
| --- | --- | --- |
| LASER | AAGGTGCCACAGATGCTCAA | GGGAGGTATCCCGGAGAAGT |
| GAPDH | GAAGGTGAAGGTCGGAGTC | GAAGATGGTGATGGGATTTC |
| MTTP | ACGGCCATTCCCATTGTG | GCCAGAGCTCCGAGAGAGAA |
| APOB | CTGCAGCTTCATCCTGAAGA | CAGGATGTAAGTAGGTTCATC |
| SREBP1 | CATTGAGCTCAAGGATCTGG | GTCCTCCACCTCAGTCTTCAC |
| HMGCR | GCAGCAAACATTGTCACCG | CACCACCCACCGTTCCTAT |
| PCSK9 | CCAAGCCTCTTCTTACTTCACC | GCATCGTTCTGCCATCACT |
| FAS | TGTGGACATGGTCACGGAC | GGCATCAAACCTAGACAGGTC |
| LSD1 | TGACCGGATGACTTCTCAAGA | GTTGGAGAGTAGCCTCAAATGTC |
| EZH2 | AATCAGAGTACATGCGACTGAGA | GCTGTATCCTTCGCTGTTTCC |
| MYLIP | TCTCCTCTGCCACCTTGAAC | TCCATTGCCGACACAATCTG |
| ABCA1 | TTCCCGCATTATCTGGAAAGC | CAAGGTCCATTTCTTGGCTGT |
| HNF-1α | TGGCGCAGCAGTTCACCCAT | TGAAACGGTTCCTCCGCCCC |
| LXR | CCTTCAGAACCCACAGAGATCC | ACGCTGCATAGCTCGTTCC |
| HNF-1α CHIP primer | GAGCCCAGATCATGCCATTG | AAAAGGGCGAGTTTGATGGT |
| PCSK9 CHIP primer | TCCAGCCCAGTTAGGATTTG | CGGAAACCTTCTAGGGTGTG |

**Supplementary Figure 1**


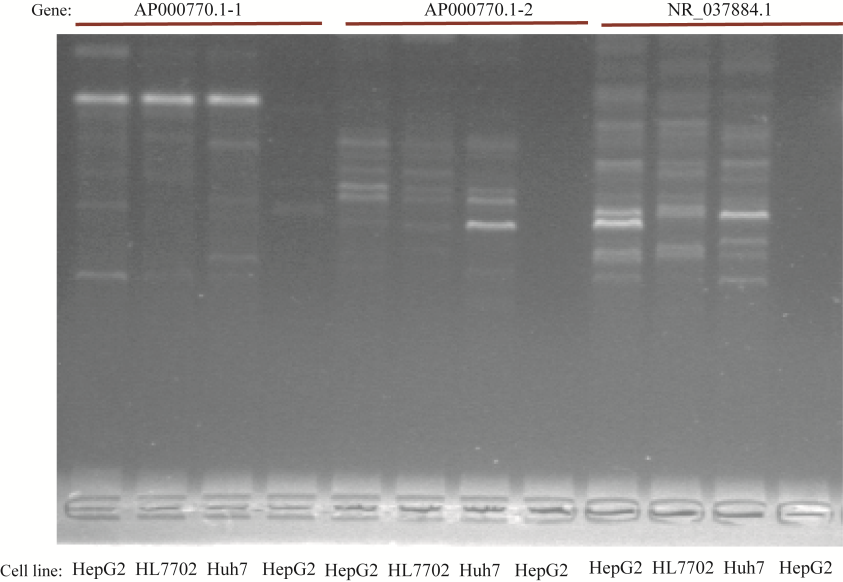

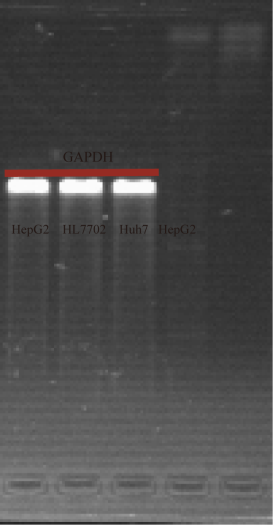


**Supplementary Figure 1**. Identification of LASER in hepatocyte cell lines, and the association of LASER with cholesterol levels in PBMCs from patients.

Full-length gels of Figure 1A.The expressions of AP000770.1-1, AP000770.1-2 and NR_037884.1 in different cell lines were determined by semi-quantitative RT-PCR. The amplified products were visualized following 2% agarose gel electrophoresis. To verify the specificity of RT-PCR, one negative control is shown without reverse transcriptase.

**Supplementary Figure 2**


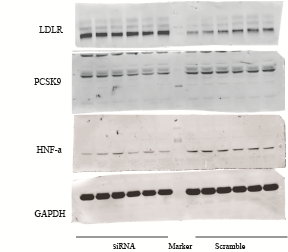


**Supplementary Figure 2**. The protein levels of HNF-1α, PCSK9, LDLR and GAPDH were determined by western blotting after siRNA (50 nM) against LASER or scramble control

Full-length blots of Figure 2J. The expressions of HNF-1α, PCSK9, LDLR and GAPDH in HepG2 were determined by western blotting.
